# Polycomb repressive complex 1 modulates granulosa cell proliferation in early folliculogenesis to support female reproduction

**DOI:** 10.7150/thno.89878

**Published:** 2024-01-27

**Authors:** Meng Gao, Tuo Zhang, Tengxiang Chen, Ziqi Chen, Zijian Zhu, Yang Wen, Shaogang Qin, Yibing Bao, Ting Zhao, Hengxing Li, Longping Liu, Ming Hao, Jianbin Wang, Fengchao Wang, Haibin Wang, Bo Zhou, Hua Zhang, Guoliang Xia, Chao Wang

**Affiliations:** 1 State Key Laboratory of Farm Animal Biotech Breeding, College of Biological Sciences, China Agricultural University, Beijing 100193, China.; 2 Guizhou Provincial Key Laboratory of Pathogenesis & Drug Research on Common Chronic Diseases, Transformation Engineering Research Center of Chronic Disease Diagnosis and Treatment, Department of Physiology, College of Basic Medicine, Guizhou Medical University, Guiyang, Guizhou Province, 550025, China.; 3 State Key Laboratory of Animal Nutrition, College of Animal Science and Technology, China Agricultural University, Beijing 100193, China.; 4 School of Life Sciences, Tsinghua University, Beijing, 100084, China.; 5 Transgenic Animal Center, National Institute of Biological Sciences, Beijing, 102206, China.; 6 Fujian Provincial Key Laboratory of Reproductive Health Research, Department of Obstetrics and Gynecology, The First Affiliated Hospital of Xiamen University, School of Medicine, Xiamen University, Xiamen, Fujian Province, 361005, China.; 7 Key Laboratory of Ministry of Education for Conservation and Utilization of Special Biological Resources in Western China, College of Life Science, Ningxia University, Yinchuan, 750021, China.

**Keywords:** Folliculogenesis, PRC1, CDKI, granulosa cell proliferation, infertility, POI

## Abstract

**Rationale:** Premature ovarian insufficiency (POI) is an accelerated reduction in ovarian function inducing infertility. Folliculogenesis defects have been reported to trigger POI as a consequence of ovulation failure. However, the underlying mechanisms remain unclear due to the genetic complexity and heterogeneity of POI.

**Methods:** We used whole genome sequencing (WGS), conditional knockout mouse models combined with laser capture microdissection (LCM), and RNA/ChIP sequencing to analyze the crucial roles of polycomb repressive complex 1 (PRC1) in clinical POI and mammalian folliculogenesis.

**Results:** A deletion mutation of *MEL18*, the key component of PRC1, was identified in a 17-year-old patient. However, deleting *Mel18* in granulosa cells (GCs) did not induce infertility until its homolog, *Bmi1*, was deleted simultaneously. Double deficiency of BMI1/MEL18 eliminated PRC1 catalytic activity, upregulating cyclin-dependent kinase inhibitors (CDKIs) and thus blocking GC proliferation during primary-to-secondary follicle transition. This defect led to damaged intercellular crosstalk, eventually resulting in gonadotropin response failure and infertility.

**Conclusions:** Our findings highlighted the pivotal role of PRC1 as an epigenetic regulator of gene transcription networks in GC proliferation during early folliculogenesis. In the future, a better understanding of molecular details of PRC1 structural and functional abnormalities may contribute to POI diagnosis and therapeutic options.

## Introduction

Premature ovarian insufficiency (POI) is one of the ovarian failures that is clinically characterized by amenorrhea with hypoestrogenic and hypergonadotropic conditions before the age of 40 years 1. Women with POI are generally infertile, along with multiple long-term health risks, such as osteoporosis and cardiovascular disorders 1. Approximately 110 POI-causative genes have been identified based on either a physiological role in ovaries or the phenotype of mutant mouse models 2. However, variants of these genes only explain a small fraction of POI due to the complicated and heterogeneous pathogeny 2-4. Additional potential genetic candidates need to be investigated to identify the underlying molecular mechanisms of POI.

Mammalian folliculogenesis is a complex but well-organized process, including oocyte growth, maturation, and proliferation and differentiation of surrounding granulosa cells (GCs) 5, 6. Upon activation of primordial follicles (PrFs) into primary follicles (PFs), the flattened pre-GCs transform into cubical GCs, initiating oocyte growth. Subsequently, the oocyte growth is accompanied by rapid GC proliferation, and the follicles develop into secondary follicles (SFs) 7. Under the cyclical induction of gonadotropins, GCs differentiate into cumulus cells (CCs) and mural granulosa cells (MGCs), accelerating oocyte maturation 1, 8, 9. Besides maintaining a stable follicular microenvironment, GCs support oocyte development by providing signals, such as KIT ligand (KITL), C-type natriuretic peptide (CNP), and metabolites, such as cholesterol, pyruvic acid and specific amino acids 10-15. Thus, the development of GC plays an indispensable role in folliculogenesis and oocyte maturation. Importantly, the defect in folliculogenesis is recognized as one of the causes of POI 16. As known, a class of mutations occurring in GC-specific genes are related to POI. These genes, including forkhead box L2 (*FOXL2*), follicle stimulating hormone receptor (*FSHR*) and hyaluronan mediated motility receptor (*HMMR*), regulate GC development to maintain female reproduction, 2, 17-23. Although growth differentiation factor 9 (GDF9) is one of the oocyte-secreted factors (OSFs), it regulates GC proliferation during the primary-to-secondary transition to maintain female fertility 1, 24, 25. Despite genetic mutations that modulate GC development crucial for POI, additional potential candidates of POI causative molecules need to be investigated.

Canonical polycomb repressive complex 1 (PRC1) in mammals consists of two core components, the catalytic subunits RING1A and its paralogue RING1B, and the structural subunits BMI1 or MEL18 26, 27. PRC1 acts as a transcriptional repressor by catalyzing mono-ubiquitination of histone H2A at lysine 119 (H2AK119ub1), which directly suppresses gene transcription by enriching on the promoters 28, 29. In several studies, the deletion of *Ring1a/b* eliminated PRC1 functions, evidenced by the subsequent decrease in H2AK119ub1 30-32. In oogenesis, *Ring1a/b* deletion leads to the loss of H2AK119ub1, which disturbs meiosis initiation by elevating *Stra8* expression in primordial germ cells or damages the meiosis arrest by promoting the transcription of meiotic prophase-I genes in oocytes of PFs 30, 32. In addition, PRC1 structural subunits are responsible for female reproduction in multiple ways. For instance, BMI1 regulates progesterone receptor ubiquitination in the uteruses, essential for embryo implantation in mice and humans 33. Furthermore, GC-specific depletion of* Mel18*, a homologue of *Bmi1*, leads to an early onset of infertility by ovulation defects in 10-month-old mice 34.

We identified a *MEL18* genetic mutant in an adolescent POI patient, suggesting that MEL18 is one of the genetic candidates related to POI. Based on mouse models, deficiency of BMI1 on a background lacking MEL18 in GCs triggered severe reproductive deficiencies, such as GC proliferation failure, follicular blockage, and complete loss of fertility. Double deletion of *Bmi1*/*Mel18* eliminated PRC1 catalytic function, decreasing H2AK119ub1, which elevated cyclin-dependent kinase inhibitors (CDKIs) levels to block the cell cycle at the G1-S phase. We demonstrated that PRC1, as an epigenetic regulator, controls gene transcription networks to coordinate GC proliferation in mice, which is crucial for early folliculogenesis and female reproduction.

## Methods

### Patients

In this study, we evaluated one affected family with premature ovarian insufficiency who were recruited from the Affiliated Hospital of Guizhou Medical University, China. The human studies were approved by the College of Basic Medicine, Guizhou Medical University (IRB No. 2022-46), and the sample were donated voluntarily for scientific research after informed consent was obtained.

### Mice

*Bmi1*-floxed mouse line (*Bmi1^fl/fl^*) and *Mel18*-floxed mouse line (*Mel18^fl/fl^*) were provided by Prof. Rongwen Xi (National Institute of Biological Sciences, Beijing, China) and constructed as previously described 35. GC-specific single or double knockout mice were generated by mating *Bmi1^fl/fl^*,* Mel18^fl/fl^* and *Bmi1^fl/fl^/Mel18^fl/fl^* females with *Foxl2-Cre* males respectively, which were kindly donated by Prof. Fengchao Wang. All mice were maintained on identical C57BL/6J genetic backgrounds. Genotyping primers are presented in Table S1.

Mice were housed in mouse facilities under 12/12-h light/dark cycles at 26°C and 40-70% humidity with access to chow and water ad libitum, according to the guidelines for the care and use of laboratory animals. All procedures were conducted according to the guidelines approved by the Animal Research Committee of the China Agricultural University, No. AW81601202-3-12.

### Mouse fertility and ovulation assay

For the mice fertility test, a 6-week-old dcKO female and its littermate wild-type (WT) female were housed with a 2-month-old WT male with normal fertility. Each WT male mouse was housed with one dcKO and one WT female mouse simultaneously. Mating cages were monitored daily. The number of pups (both alive and dead) was counted on the first day of delivery. The mating process lasted for 6 months.

For superovulation, 21 days post parturition (dpp) dcKO and WT mice were intraperitoneally injected with 5 IU PMSG (Ningbo Sansheng Biological Technology, Cat#110251283), followed by 5 IU hCG (Ningbo Sansheng Biological Technology, Cat#110041282) 44 h later. After an additional 12 h, oocytes of each mouse were collected from oviducts, and the number of oocytes was counted after digesting with 0.3% hyaluronidase (Merck, Cat#MR-051-F).

### Hormone assays

Mice at random stages of the estrous cycle were anesthetized with pentobarbital sodium solution at 80 mg/kg (body weight), and blood was collected by retro-orbital bleeding. Collected blood was allowed to clot at 4°C overnight before centrifugation at 3,000 rpm for 5 min for serum separation. Sera were stored at -80°C until assayed. Hormone levels were measured by The Reproductive Medicine Center, Department of Obstetrics and Gynecology, The Affiliated Hospital of Guizhou Medical University (Guiyang, China). The assays were performed in the Cobas e602 module of the Cobas 8000 total automation system (Roche).

### Ovary culture *in vitro*

ICR mice were obtained from Beijing Vital River Laboratory Animal Technology Co., Ltd. and housed at China Agricultural University. Mice with a vaginal plug in the next morning of mating were considered as 0.5 days post coitum (dpc), and the day after birth was considered as 1 dpp. Undamaged ovaries of 3 dpp mice were micro-dissected in cold phosphate-buffered saline (PBS) under a microscope. Isolated ovaries were cultured on 0.4 µm pore size inserts (Millipore, Cat#PICM0RG50) in 6-well culture plates (NEST, Cat#703002) for 4 days. Basal culture medium comprised 3 mL Dulbecco Modified Eagle Medium/Ham (DMEM)-F12 nutrient mixture (Gibco, Cat#C11330500BT) supplemented with penicillin-streptomycin (1:100, Gibco, Cat#15140-122). As a control, ovaries were treated with AIL (10nM, Selleck, Cat# S6885) or DMSO (Sigma, Cat#344282). Approximately half of the medium in each well was replaced with fresh medium every other day. Ovaries were maintained at 37°C under 5% CO2 and 95% air.

### Follicle counting and GC characterization

Fresh ovarian samples were fixed in 4% paraformaldehyde (Santa Cruz, Cat#30525-89-4) overnight, embedded in paraplast (Leica, Cat#39601095), and sectioned serially at 5 μm (before 7 dpp) or 8 μm (after 7 dpp). Tissue sections were stained with hematoxylin (Solarbio, Cat#G4070) to count the number of follicles. Sections were examined and photographed using VENTANA DP200 (Roche).

We classified the follicle stage by its morphological characteristics 36. A PrF contained an oocyte with a diameter less than 20 μm and one-layer flattened pre-GCs; A PF contained a larger oocyte and one-layer cubical GCs; A SF had multilayer GCs, an abPF contained an oocyte with a diameter larger than 40 μm and only one-layer cubical GCs; AFs had antral cavities.

The number of every follicle stage in 5-μm ovarian sections was counted in every fifth section and multiplied by 5 to calculate all follicles in each ovary. In 8-μm ovarian sections, the number of every follicle stage was summarized by counting all sequential sections. In both cases, only follicles containing clearly visible oocyte nucleus in each individual section were counted to avoid repetitive counting.

For characterizing GCs, the follicle was measured at the maximum cross-section with a clear oocyte nucleus. The oocyte diameter was measured by VENTANA ImageViewer v.3.2.0 (Roche). In the same follicle, the layer/thickness of the GCs was acquired by averaging the maximum and minimum number/thickness of layers by VENTANA ImageViewer v.3.2.0 (Roche).

The percentage of growing follicle number was divided by the total follicle number per ovary to obtain the follicular activation rate.

### Immunostaining and biological assays

Ovarian paraffin sections were deparaffinized, rehydrated, and subjected to high-pressure antigen repair with 0.01% sodium citrate buffer (pH=6.0) for 20 min. Immunohistochemistry assay was performed using Histostain™-SP Kits (ZSGB-BIO, Cat#PV-9001) and DAB peroxidase substrate kits (ZSGB-BIO, Cat#ZLI-9017) according to the manufacturer's protocols. Nuclei were stained with hematoxylin (Solarbio, Cat#G4070). Primary antibodies and dilution rates were as follows: rabbit anti-BMI1 (1:200, Cell Signaling Technology, Cat#D20B7); goat anti-MEL18 (1:200, Abcam, Cat#ab5267); rabbit anti-Cyclin E1 (1:100, Proteintech, Cat#11554); rabbit anti-Cyclin E2 (1:200, Proteintech, Cat#11935). Sections were examined and photographed using VENTANA DP200 (Roche).

For immunofluorescence assay, ovarian paraffin sections were deparaffinized, rehydrated, and subjected to high-pressure antigen repair with 0.01% sodium citrate buffer (pH=6.0) for 20 min. The sections were then rinsed thoroughly with PBS for 10 min and blocked with 10% normal donkey serum (Yesean, Cat#36116ES10) in PBS for 1 h at room temperature and incubated with primary antibodies (diluted with PBS) for 16 h at 4°C. Primary antibodies and dilution rates are as follows: mouse anti-DDX4 (1:400, Abcam, Cat#ab27591); goat anti-FOXL2 (1:400, Novus, Cat#NB100-1277); rabbit anti-H2AK119ub1 (1:1000, Cell Signaling Technology, Cat#8240); rabbit anti-CX43 (1:200, Abcam, Cat#ab11370); rabbit anti-Radixin (1:200, Abcam, Cat#ab52495); rabbit anti-SMAD3 (1:200, Cell Signaling Technology, Cat#9523); rabbit anti-ALK5 (1:200, Proteintech, Cat#30117); rabbit anti-BMPR2 (1:200, Proteintech, Cat#19087); rabbit anti-p21 (1:200, Abcam, Cat#188224); rabbit anti-p27 (1:200, Abcam, Cat#ab32034); rabbit anti-p-p27 (1:200, Abcam, Cat#ab62364); rabbit anti-Ki67 (1:400, Cell Signaling Technology, Cat#D385); sheep anti-BrdU (1:400, Abcam, Cat#ab1893); rabbit anti-p-H3 (1:200, Abcam, Cat#ab32107); rabbit anti-p-RB (1:200, Cell Signaling Technology, Cat#D20B12); rabbit anti-CDK2 (1:200, Abcam, Cat#ab32147); rabbit anti-γH2AX (1:200, Cell Signaling Technology, Cat#80312); rabbit anti-C-caspase3 (1:200, Beyotime biotechnology, Cat#AC033). Next, ovarian sections were rinsed thoroughly with PBS for 1 h and incubated with Alexa Fluor 488- or 555- conjugated secondary antibody (1:200, Yesean, Cat#33106ES60) for 1 h at 37°C. Subsequently, the sections were again rinsed thoroughly with PBS, stained with Hoechst33342 (1:100, Sigma, Cat#14533) for 1 min, and sealed in anti-fade fluorescence mounting medium (Applygen, Cat#C1210) with microscope cover glass (Citoglas, Cat#10212450C). Sections were examined and photographed using a Nikon A1 Confocal microscope.

For the *in vivo* BrdU assay, 5-BrdU (100 μg/g, Sigma, Cat#HY-15910) was intraperitoneally injected into 3-week-old mice and sampled the ovaries after 1 h. For the *in vitro* assay, 5-BrdU (10 μM) was added into the culture medium and sampled the ovaries after 1 h. The next steps to label positive GCs at the S phase were similar to the immunofluorescence protocols described above.

For Ki67/BrdU/p-H3-positive GC analysis, the percentage was quantified as the number of GCs with positive signal divided by the total number of GCs per maximum follicular cross-section in the abnormal primary follicles (abPFs) of dcKO and the SFs of WT mice. Sections were examined and photographed using a Nikon A1 Confocal microscope.

Apoptosis signals were detected by the TUNEL BrightGreen Apoptosis Detection Kit (Vazyme, Cat#A112-03) in ovarian paraffin sections following the manufacturer's recommendation. Sections were examined and photographed using a Nikon A1 Confocal microscope.

Cell senescence signals were detected by Senescence β-Galactosidase Staining Kit (Beyotime Biotechnology, Cat#C0602) in 8-μm ovarian frozen sections. The nuclear fast red staining solution (Beyotime biotechnology, Cat#C0151) was used to label nuclei. Sections were examined and photographed using VENTANA DP200 (Roche).

TZPs were labeled by using phalloidin to mark filamentous actin. Eight-μm ovarian frozen sections were first blocked for 1 h with 5%BSA in PBST (PBS plus 0.1% Triton) and then incubated with Phalloidin-TRITC (1:1000, Thermo Fisher Scientific, Cat#R415) for 1 h at room temperature. Sections were examined and photographed using a Nikon A1 Confocal microscope.

### Transmission electron microscopy (TEM)

3-week-old WT and dcKO ovaries were fixed in 2.5% glutaraldehyde overnight at 4°C. Then, the treated ovaries were processed and wrapped in epoxypropane resin following standard TEM procedures. Subcellular structures were observed in the abPFs of dcKO and the early SFs of WT mice with similar oocyte diameters.

### RT-qPCR

Total RNA was isolated from ovaries with TRIzol (Invitrogen, Cat#15596018). The quantity and quality of total RNA were determined using Nanodrop (Thermo Fisher Scientific). 1 μg total RNA of each sample was used to reverse transcribe into cDNA according to manufacturer's recommendation (Takara, Cat#RR047A). RT-qPCR was performed in 96-well plates (Roche, Cat#04729692001) using FastStart Universal SYBR^®^ Green Master (Roche, Cat#61396600) with LightCycler^®^ 96 Real-Time PCR System (Roche). Reaction parameters were as follows: 10 min at 95°C, followed by 45 cycles of 10 s at 95°C and 30 s at 60°C. Data were normalized to *Actb*. Primers are presented in Table S1.

### Western blotting

Total protein from ovaries was extracted with TRIzol (Invitrogen, Cat#15596018), separated on 10% SDS-PAGE, and transferred to PVDF (polyvinylidene fluoride) membranes (Millipore, Cat# IPVH00). The membranes were blocked in 5% skim milk (Solarbio, Cat#D8340) for 1 h at room temperature and incubated with relevant primary antibodies (diluted with TBST (TBS plus 0.05% Tween-20)) overnight at 4°C. After rinsing with TBST, the membranes were incubated with the HRP-linked secondary antibody (1:4,000, ZSGB-BIO, Cat#ZB-2301/ZB-2305) for 1 h at room temperature and rinsed again with TBST. The membranes were visualized using the SuperSignal detection system (Thermo Fisher Scientific, Prod 34080). the image was quantified using Adobe Photoshop CS6. Primary antibodies and dilution rates were as follows: rabbit anti-α-Tubulin (1:1000, Beyotime biotechnology, Cat#AF0001); rabbit anti-H3 (1:1000, Abcam, Cat#ab1791); rabbit anti-GAPDH (1:1000, Proteintech, Cat#10494); rabbit anti-DDX4 (1:1000, Abcam, Cat#13840); goat anti-FOXL2 (1:500, Novus, Cat#NB100-1277); rabbit anti-BMI1 (1:500, Cell Signaling Technology, Cat#6964); rabbit anti-MEL18 (1:1000, provided by Prof. Qun He); rabbit anti-H2AK119ub1 (1:500, Cell Signaling Technology, Cat#8240); rabbit anti-GDF9 (1:500, Abcam, Cat#ab38544); rabbit anti-SMAD3 (1:500, Beyotime biotechnology, Cat#AF1501); rabbit anti-p-SMAD3 (1:500, Cell Signaling Technology, Cat#C25A9); mouse anti-p-RB (1:500, Santa Cruz, Cat#377528); mouse anti-E2F1 (1:500, Santa Cruz, Cat#251); rabbit anti-PCNA (1:500, Beyotime biotechnology, Cat#AF1363); rabbit anti-Caspase-3 (1:500, Cell Signaling Technology, Cat#9662).

### Whole genome sequencing (WGS) and variant analysis

WGS was performed using peripheral blood from the patient and her normal sister by Frasergene Co., Ltd. (Wuhan, China). Copy number variations (CNV) were detected by CNVnator v0.4.1 and annotated using AnnotSV V1.1.1.

### Ovary RNA-seq

2-week-old ovaries of WT and dcKO mice were collected, and RNA was extracted by TRIzol (Invitrogen, Cat#15596018). cDNA libraries were sequenced on the NovaSeq 6000 Illumina sequencing platform and analyzed by Novogene Co., Ltd. (Beijing, China). Differentially expressed genes (DEGs) were analyzed using the DESeq2 R package (1.20.0). Generally, genes with *P* values less than 0.05 and absolute fold-change larger than 2 were considered DEGs.

### laser capture microdissection (LCM)-associated RNA-seq

Ovaries from 3-week-old dcKO and WT were cut into 8-µm frozen sections and mounted onto cross-linked polyethylene (PEN) foil attached to a glass slide (Leica, Cat#11505158) to facilitate the removal of selected specimens from the slides into PCR tubes. Slides were then processed for Crystal Violet (Sigma, Cat#C0775) staining. An LMD microdissection system (Leica, LMD 7000) was used to capture oocyte tissues and GCs into 0.2 mL PCR tubes by utilizing a pulsed UV laser to melt the foil film. All samples were stored at -80°C for RNA extraction.

The cDNA library of the samples was built following the protocol by Picelli et al. 37. After purification, the samples were sequenced on the NovaSeq 6000 Illumina sequencing platform. Subsequently, the DEG analysis was performed using the DESeq2 R package v.1.20.0. Generally, genes with *P* values less than 0.05 and absolute fold-change larger than 4 were considered DEGs.

### ChIP-seq

ChIP-seq was performed to identify H2AK119ub1-enriched regions within the whole genome. To isolate GCs, about 60 ovaries of 10-12 dpp ICR mice per group were completely digested with 0.25% Trypsin (Gibco, Cat#25200-702) at 37°C. The digestion was stopped by adding the same volume of serum (Gibco, Cat#10099-141), and the single-cell suspension was transferred into a 40 μm cell strainer (Biologix, Cat#15-1040) to remove oocytes and centrifuged at 3,000 rpm for 5 min to collect GCs. The ChIP-seq library was sequenced on the Illumina sequencing platform and analyzed by Novogene Co., Ltd. (Beijing, China). ChIPseeker was used to retrieve the nearest genes around the peak and annotate the genomic region of the peak. Gene visualization was carried out by Integrative Genomics Viewer v.2.16.1.

### ChIP-qPCR

GCs from 10-12 dpp ICR ovaries were collected using the above-mentioned method. ChIP assays were performed using the SimpleChIP Enzymatic Chromatin IP Kit (Cell Signaling Technology, Cat#9003) according to the manufacturer's protocol. Immunoprecipitations were performed with cross-linked chromatin from the GCs and rabbit anti-H2AK119ub1 (1:25, Cell Signaling Technology, Cat#8240) or rabbit IgG (1:25, Abcam, Cat#ab172730). Purified DNA was quantified by RT-qPCR using the above-mentioned reaction parameters. Data were normalized to the Input (2%) group. RT-qPCR products were mixed with Gel Loading Dye (NEB, Cat#B7024S) and identified by 1.5% agarose (Biowest, Cat#111860) gel electrophoresis using the Direct-Load Star Marker D2000 (GenStar, Cat#M122). Primers are shown in Table S1.

### Statistical analysis

All experiments were repeated at least three times. Data were presented as the mean values ± Data were expressed as the means ± standard error of the mean (SEM) of each experiment. Data were analyzed by Student's t-test and were considered statistically significant at * (*P* < 0.05), ** (*P* < 0.01), *** (*P* < 0.001) and not significant (n.s.) at *P* ≥ 0.05. Statistics and graphs were obtained using GraphPad Prism v.9.3.1.

## Results

### *MEL18* mutation was related to POI in women

**Figure 1:**
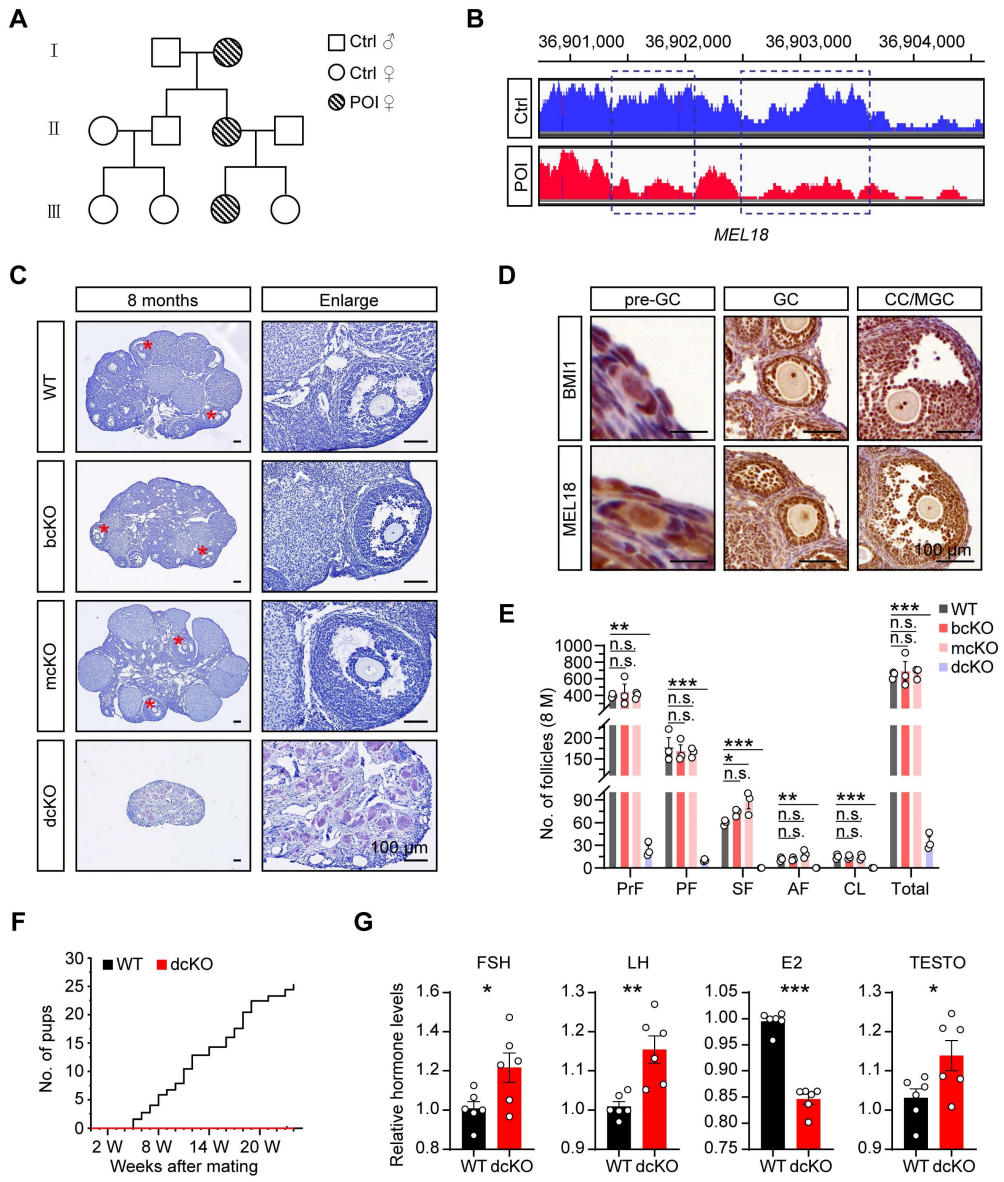
** BMI1/MEL18 coordinated to regulate female reproduction in mice.** (**A**) Pedigrees of the family. Horizontal lines represent the unions. (**B**) CNV analysis of *MEL18* in the patient and her normal sister. Dotted rectangles mark the deletion mutation regions. (**C**) Hematoxylin staining of 8-month-old ovaries. Asterisks mark AFs. (**D**) Immunohistochemical staining of BMI1 (brown, top) and MEL18 (brown, bottom) in 3-week-old WT ovaries. (**E**) Number of follicles in 8-month-old ovaries. n = 3. Total, all follicle types. (**F**) Cumulative number of pups from WT (n = 3) and dcKO (n = 6) female. (**G**) Relative hormone levels of FSH, LH, E2 and TESTO in 8-month-old mice serum. n = 6.

To identify the genetic causes of POI, we systematically analyzed the basic information of a consanguineous family in which each generation had a POI female. Among them, the 17-year-old patient (III) was amenorrheal with higher follicle-stimulating hormone (FSH), luteinizing hormone (LH), and lower estradiol (E2) levels (Figure 1A, S1A). The patient had a normal-sized uterus (34×24×29 mm in size) but smaller ovaries (left:17×5 mm, right:13×7 mm) than average (approximately 40×30 mm in adults) (Figure S1A), suggesting that the ovarian folliculogenesis was impaired due to a genetic mutation. The karyotype analysis showed no chromosomal abnormality (Figure S1B). However, the CNV analysis according to the WGS data revealed 744 variants in the patient's genome compared with her normal sister. Specifically, we noticed a large fragment deletion mutation of *MEL18* on the chromosome, which had never been reported before (Figure 1B). The findings implied that *MEL18* may be one of the candidates responsible for POI.

### Co-deletion of *Bmi1*/*Mel18,* not *Mel18* alone, caused POI-like defects in mice

We explored the role of MEL18 in ovaries by examining its expression pattern during folliculogenesis. The results showed that MEL18 was expressed in oocytes and somatic cells of all developmental stages (Figure 1D). Furthermore, MEL18 expression was observed in flattened pre-GCs, cubical GCs, and differentiated CCs and MGCs.

Based on the report that *Mel18*-null mice die around 4 weeks after birth because of growth retardation 38, we utilized conditional knockout mice to explore the MEL18 function. *Mel18* was conditionally knocked out after 12.5 dpc using *Foxl2-Cre* mice to generate *Mel18^f/f^*; *Foxl2-Cre* (mcKO) mice (Figure S2A, F) and to evaluate its specific effect in GCs. However, unlike the severe POI defects, the morphology and number of follicles had minimal difference between 8-month-old mcKO and WT ovaries. However, the mcKO group had a larger number of SFs than WT, probably reflecting impaired development of SFs induced by the ovulation defects 34 (Figure 1C, E). Meanwhile, BMI1, the MEL18 homolog 35, 39, 40, exhibited a similar expression pattern in ovaries (Figure 1D), indicating that simultaneous deletion of *Bmi1*/*Mel18* in GCs may be necessary to eliminate their functional redundancy. Notably, *Bmi1^f/f^ Mel18^f/f^*; *Foxl2-Cre* (dcKO) mice were infertile during the 6-month mating process (Figure 1F). Similar to the characteristics of POI, dcKO ovaries in 8-month-old mice were much smaller in size than the WT group, and with almost no growing follicles (Figure 1C, E). Moreover, aberrant hormone levels, including elevated FSH, LH, and testosterone (TESTO) and reduced E2, were detected in dcKO mice sera (Figure 1G). Consistent with the mcKO mice, 8-month-old* Bmi1^f/f^*; *Foxl2-Cre* (bcKO) ovaries displayed normal folliculogenesis (Figure 1C, E).

Thus, deleting *Mel18* alone did not trigger POI-like reproduction defects in mice unless *Bmi1* was also lacking simultaneously.

### Dysfunctional PRC1 blocked early folliculogenesis at the primary-to-secondary stage

**Figure 2:**
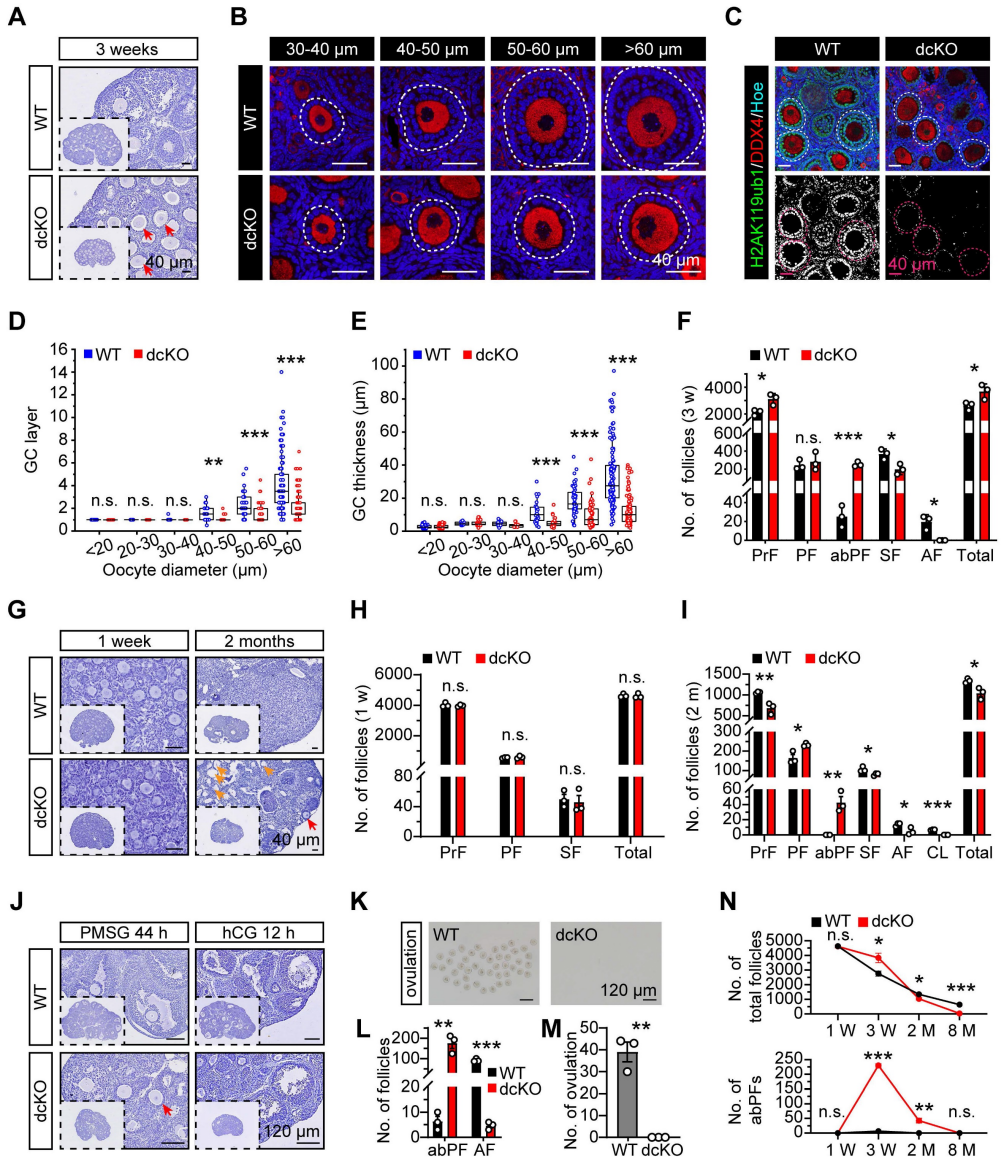
** PRC1 regulated early folliculogenesis by controlling the transition from PFs to SFs.** (**A, G**) Hematoxylin staining of 1-week, 3-week and 2-month-old ovary sections. Red arrows mark abPFs. Orange arrows mark atretic follicles. (**B**) DDX4 staining (red) and nucleus with Hoechst (blue) staining showing the follicular development in 3-week-old ovaries. Dotted lines mark follicles. (**C**) H2AK119ub1 staining (green) in 3-week-old ovaries. Dotted lines mark follicles. Images with the same threshold are on the right. (**D, E**) GC layers and thickness of follicles in 3-week-old ovaries. n = 301 follicles (WT), n = 324 follicles (dcKO). (**F, H, I**) Number of follicles in 3-week, 1-week and 2-month-old ovaries. n = 3. (**J**) Hematoxylin staining of 3-week-old ovaries after gonadotropins injection. Arrow marks abPF. (**K, M**) Morphology and number of ovulated oocytes. n = 3. (**L**) Number of abPFs and AFs on PMSG 44 h. n = 3. (**N**) Number of total follicles and abPFs at specific timepoint. n = 3.

We used 3-week-old mouse models to investigate BMI1/MEL18 functions in female reproduction and assess folliculogenesis. In dcKO ovaries, both BMI1 and MEL18 were deleted efficiently, along with a decreased H2AK119ub1 level, suggesting that the stability and the catalytic activity of PRC1 were obliterated (Figure 2C, S2F). In contrast, H2AK119ub1 levels in bcKO and mcKO ovaries were similar to those of the WT group (Figure S2F, G). Therefore, only a double deficiency of BMI1/MEL18 in GCs impaired PRC1 function and induced reproductive defects in mice.

In dcKO ovaries, the growth of oocytes and proliferation of GCs were out-of-sync in a large fraction of growing follicles; the oocytes grew to the SF stage while the GC proliferation was apparently delayed (Figure 2A). As expected, no apparent developmental abnormalities were observed in either bcKO or mcKO ovaries (Figure S2H, I). To further investigate the causes of follicular developmental defects in dcKO mice, the preantral follicles were categorized into six groups based upon the oocyte diameters of <20 μm, 20-30 μm, 30-40 μm, 40-50 μm, 50-60 μm, and >60 μm and the differences in the GC development between WT and dcKO mice were compared. In WT mice, follicles in the <20 μm group were dormant PrFs, while in the 30-40 μm group were uniformly PFs. Notably, once the oocyte diameter was larger than 40 μm, the GCs proliferated to form the second layer and reached the SF stage (Figure 2D). However, the GC development of follicles in dcKO was impaired when the oocyte diameter developed to 40-50 μm, in which the GCs remained unilaminar, and the GC layer was much thinner than in the WT group (Figure 2D, E). Additionally, the GC morphology in these follicles was cuboidal or columnar, suggesting that the activation from PrFs to PFs was normal in dcKO mice (Figure 2B). We, therefore, named these impaired follicles as abnormal primary follicles (abPFs) to distinguish them from normal PFs. Importantly, there was a significant shortage of SFs and AFs in 3-week-old dcKO ovaries, possibly due to the blockage of follicles at the abPF state (Figure 2F).

Folliculogenesis of dcKO mice was systematically evaluated by examining the follicle development at several time points. Initially, 19.0 dpc ovaries were collected to determine if BMI1/MEL18 regulated PrF formation or *Foxl2-Cre* affected ovarian development before birth. The PrF formation was undisturbed in dcKO ovaries in which *Bmi1* and *Mel18* were deleted (Figure S2B, E), suggesting that the Cre-LoxP system worked specifically and efficiently. Moreover, we found that the H2AK119ub1 levels in ovaries and pre-GCs were similar to those in the WT group (Figure S2C-E), implying that PRC1 function was not impaired. Further, the morphology and follicle numbers in 1-week-old ovaries were similar between dcKO and WT mice, indicating that PF formation was not affected by gene deletion (Figure 2G, H).

Compared with the WT mice, in which abPFs were restrained at a low level (Figure 2N), the number of abPFs was significantly higher in 2-month-old dcKO ovaries along with decreased SFs (Figure 2G, I). Although there were elevated FSH and intact LH levels in dcKO serum, fewer AFs were present and CLs were not detected in the 2-month-old dcKO ovaries (Figure 2I, S2N). These results suggested that the follicles in dcKO failed to respond to gonadotropin stimulation, which was further corroborated by the increased number of atresia follicles and reduced level of E2 in the serum (Figure 2G, S2N). These observations indicated that compared to the WT group, the growing follicles in dcKO ovaries had a weak response to FSH induction and could not produce enough E2. Consistently, intraperitoneal injection of pregnant mare serum gonadotropin (PMSG), serving as FSH to stimulate follicle growth, failed to induce AF development, while human chorionic gonadotropin (hCG), serving as LH to induce oocyte maturation and ovulation, failed to induce ovulation in dcKO mice (Figure 2J-M).

In summary, the simultaneous deletion of *Bmi1/Mel18* abolished the PRC1 function in GCs. Dysfunctional PRC1 induced a follicle development disorder in a time-dependent manner attributed to the arrested transition of primary-to-secondary follicles (Figure 2N).

### Mutant PRC1 impaired mutual crosstalk between oocytes and GCs

**Figure 3:**
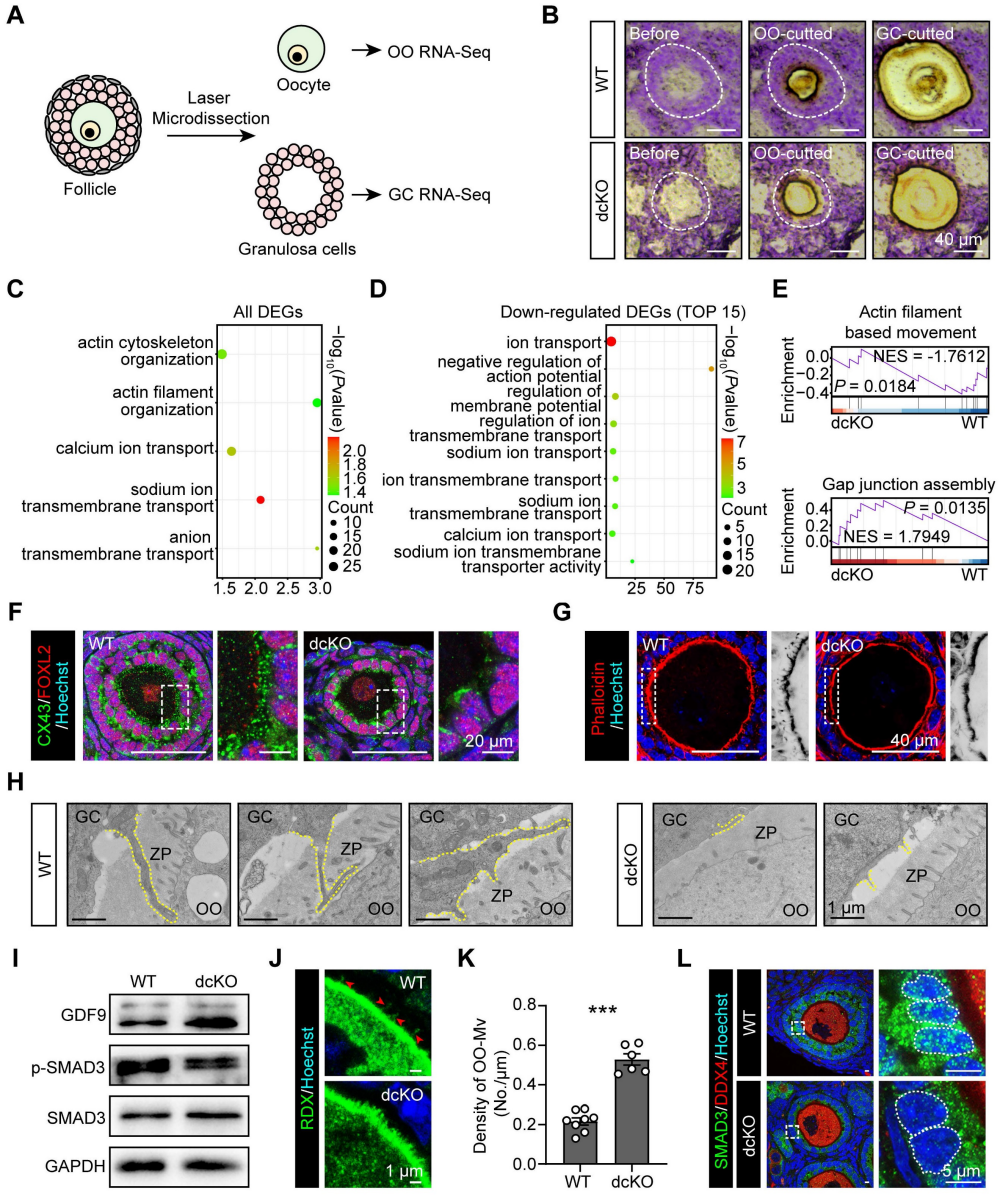
** Crosstalk in dcKO was disrupted with impaired physical connections and signaling pathways.** (**A**) Schematic diagram and (**B**) strategy of LCM assays. OO, oocyte. (**C**) Key GO enrichment of all DEGs in GCs. (**D**) TOP15 terms related to ion channels of downregulated DEGs in GCs. (**E**) GSEA of all DEGs in GCs. NES, normalized enrichment score. (**F**) CX43 staining (green) in 3-week-old ovaries. Dotted rectangles mark the areas that are enlarged on the right. (**G**) Phalloidin staining (red) in 3-week-old ovaries. Dotted rectangles mark the areas that are enlarged on the right, which are inverted to black/white to highlight OO-Mv. (**H**) TZPs (dotted lines) in 3-week-old ovaries. ZP, zona pellucida. (**I**) GDF9, SMAD3 and p-SMAD3 protein levels in 3-week-old ovaries. (**J**) RDX staining (green) in 3-week-old ovaries. Arrowheads mark the OO-Mv in the WT group. (**K**) Density of OO-Mv per follicle. n = 8 (WT), n = 6 (dcKO). (**L**) SMAD3 staining (green) in 3-week-old ovaries. Dotted rectangles mark the areas are enlarged on the right. Dotted cycle on the right marks the nucleus of a single GC.

**Figure 4:**
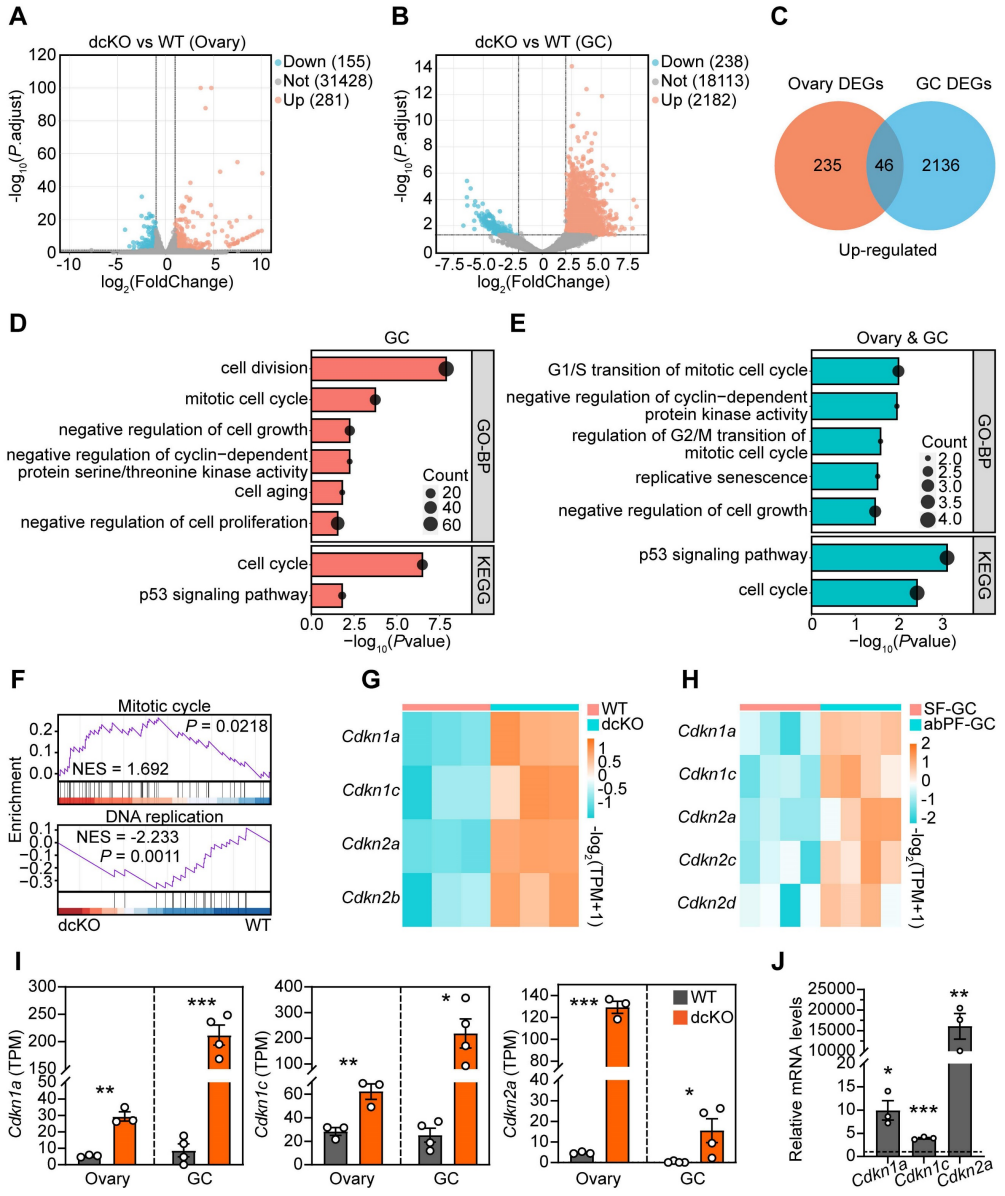
** Cell cycle of GCs was blocked in dcKO mice according to the RNA-seq analysis.** (**A**, **B**) Volcano plots showing the comparison of transcriptomes between the ovaries of WT and dcKO at 2 weeks, and between the GCs of early SFs in WT and abPFs in dcKO at 3 weeks respectively. (**C**) Venn diagrams showing the overlapping upregulated DEGs. (**D**, **E**) GO and KEGG analyses of upregulated DEGs of GCs and overlapping DEGs respectively. (**F**) GSEA of all DEGs of GCs. (**G**, **H**) Heatmaps of CDKIs according to the ovary and GC RNA-seq data respectively. TPM, transcripts per kilobase million. (**I**) TPM of *Cdkn1a*, *Cdkn1c*,* Cdkn2a*. (**J**) *Cdkn1a, Cdkn1c* and* Cdkn2a* relative mRNA levels in 3-week-old ovaries. n = 3. Dotted line indicates the relative mRNA levels of the WT group.

To identify the genes regulated by PRC1, RNA-sequencing (seq) of oocytes and surrounding GCs was performed by LCM assays. Briefly, by limiting the oocyte diameter to 40-50 μm, the oocytes and surrounding GCs of abPFs in dcKO ovaries and early SFs in WT ovaries were collected for sequencing (Figure 3A, B). Upregulation of 2,182 genes and downregulation of 238 genes was observed in the GCs of abPFs (fold change ≥ 4, *P*. adjust < 0.05, Figure 4B). Gene ontology (GO) enrichment analysis showed that the DEGs were enriched in the terms related to bidirectional communication, such as actin cytoskeleton, filament organization, and ion transport (Figure 3C). Moreover, gene set enrichment analysis (GSEA) revealed the enrichment in “actin filament-based movement” and “gap junction assembly” terms (Figure 3E). Surprisingly, the downregulated DEGs were enriched in the ion transport based on the top 15 terms, suggesting that GC-derived physical connections and communication were impaired in dcKO mice (Figure 3D).

The crosstalk network between oocytes and GCs during the transition from PFs to SFs, has been elucidated 7, 41, 42. The physical connections, including GC-derived transzonal projections (TZPs), oocyte-derived microvilli (OO-Mv), and gap junctions between oocytes and GCs, are formed in an orderly manner 43-45. We examined biomarkers of these structures based on GC RNA-seq data. Compared with the WT group, the intensity of connexin 43 (CX43) in abPFs, essential for gap junctions 46, was significantly decreased (Figure 3F). Additionally, staining of filamentous actins with phalloidin showed a noticeable lack of TZPs in abPFs (Figure 3G). TEM analysis confirmed that the TZPs nearly disappeared in abPFs, of which the GCs exhibited much weaker structures than those in WT group (Figure 3H).

We examined the key factors related to the GDF9 signaling pathway to verify the paracrine signals in abPFs 47. Strikingly, the characteristics of abPFs in this study were similar to those of the “type 3b” follicles induced by the lack of GDF9 24. We found that 1,009 genes were upregulated and 55 were downregulated in dcKO oocytes (fold change ≥ 4, *P*. adjust < 0.05, Figure S3A). Among the upregulated genes, the *Gdf9* level was increased significantly, further confirmed by the mRNA and protein expression assays (Figure 3I, S3B-D). However, levels of other OSFs, such as *Bmp15* and *Fgf8*, were not affected (Figure S3B). It has been previously reported that OO-Mv on the oocyte membrane plays a vital role in the GDF9 release 44. Thus, we verified the expression of Radixin (RDX), a specific microvillus-related protein in oocytes, and found increased OO-Mv density in the dcKO group (Figure 3J, K). Conceivably, elevated GDF9 levels, together with OO-Mv structures, might substantiate the oocyte-derived feedback regulation to promote GC proliferation. In GCs, SMAD3 in the cytoplasm is phosphorylated to p-SMAD3 by GDF9 signals and transferred to the nucleus to regulate proliferation 48, 49. However, compared with the WT group, the p-SMAD3 levels were decreased in dcKO ovaries (Figure 3I, S3D). Although the SMAD3 was elevated in the abPF GCs (Figure 3I, S3D, E), most of it was not phosphorylated and was therefore located in the cytoplasm rather than the nucleus (Figure 3L).

Our findings identified the damaged physical structures and abnormal paracrine signaling pathways in dcKO mice, emphasizing the significance of PRC1 in supporting bidirectional crosstalk during the development of early growing follicles.

### PRC1 controlled GC proliferation by regulating cell cycle progression

We performed two RNA-seq assays to screen key genes responsive to PRC1 and also important for follicle growth. One of the samples was the 2-week-old ovaries, in which the abPFs appeared initially (Figure S2J, K), while the rest sample was the GCs of specific follicles, including early SFs in WT group and abPFs in dcKO group, collected by LCM assays accordingly. We focused on the upregulated genes since the H2AK119ub1 expression level was decreased in abPFs, indicating that the gene transcription might become more active. Compared with the WT group, 281 genes (fold change ≥ 2, *P*. adjust < 0.05) were upregulated in dcKO ovaries, and 46 genes overlapped in ovaries and GCs (Figure 4A-C). By analyzing all the DEGs from GC RNA-seq data, GSEA revealed the enrichment of “mitotic cell cycle” and “DNA replication” terms (Figure 4F). In addition, GO/KEGG analyses of upregulated DEGs in GCs and common DEGs in ovaries and GCs revealed enrichment of terms related to the mitotic cell cycle and its phase transition (Figure 4D, E).

Given the significance of cell cycle-related genes in regulating mitosis, we examined their expression levels in the RNA-seq dataset. There was no apparent change in levels of cyclin-dependent kinases (CDKs) and cyclins (Figure S4A). However, mRNAs encoding various CDKIs were elevated in the dcKO group, including *Cdkn1a*, *Cdkn1c*, *Cdkn2a,* and *Cdkn2b* in ovaries and *Cdkn1a*, *Cdkn1c*,* Cdkn2a*,* Cdkn2c,* and *Cdkn2d* in the GCs of abPFs (Figure 4G, H). Among them, *Cdkn1a*, *Cdkn1c,* and *Cdkn2a* were steadily upregulated, as confirmed by RT-qPCR (Figure 4I, J). These observations indicated that dysfunctional PRC1 in GCs resulted in impaired cell cycle by upregulating CDKIs.

### PRC1 directly regulated CDKI transcription via H2AK119ub1 modification

**Figure 5:**
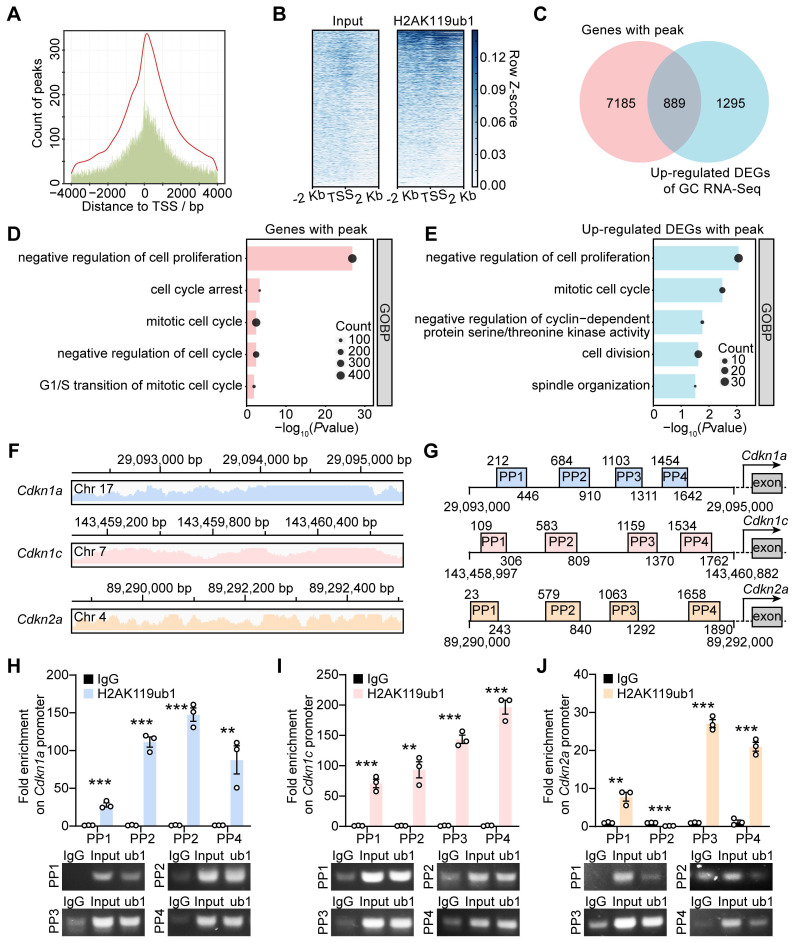
** PRC1-targeted H2AK119ub1 was responsible for repressing gene transcription of CDKIs.** (**A**) Number of H2AK119ub1 combination peaks in a ± 4 kb window of TSSs. (**B**) Read distribution of H2AK119ub1 peaks in a ± 2 kb window of TSSs. (**C**) Venn diagram showing the overlapping upregulated DEGs of GCs, of which H2AK119ub1 is enriched on the promoter. (**D**, **E**) GO analysis of H2AK119ub1-enriched genes and overlapping genes respectively. (**F**) Integrated Genomics Viewer (IGV) data showing H2AK119ub1 is enriched on the promoter of *Cdkn1a*, *Cdkn1c* and *Cdkn2a*. Chr, chromatin. (**G**) Schematic diagrams of the loci of ChIP-qPCR primers (PP1-PP4). (**H**, **I**, **J**) Enrichment degrees of H2AK119ub1 on the *Cdkn1a*, *Cdkn1c* and* Cdkn2a* promoters respectively. IgG served as the negative control and Input (2%) served as the positive control. n = 3.

Enrichment of H2AK119ub1 on the promoter region suppresses gene transcription. We performed the ChIP-seq assay using GCs from 10-12 dpp ICR mouse ovaries to determine whether the upregulated transcription of CDKIs in dcKO was directly caused by decreased H2AK119ub1 (Figure S5A). The results showed enrichment of H2AK119ub1 combined peaks in a ± 2 kb window near the transcription start sites (TSSs) of the genes (Figure 5A, B). In detail, H2AK119ub1 was enriched on the promoter regions of 8,074 genes (Figure 5C). GO analysis showed that these genes were associated with the terms “cell cycle arrest” and “negative regulation of cell proliferation/cycle” (Figure 5D). As the Venn diagram shows, a set of 889 genes was upregulated in the GCs of abPFs and identified as H2AK119ub1-targeted candidates (Figure 5C). In addition, GO analysis revealed a significant enrichment of cell cycle-related terms (Figure 5E).

The ChIP-seq data also revealed that H2AK119ub1 was significantly enriched on *Cdkn1a*, *Cdkn1c,* and *Cdkn2a* promoters (Figure 5F). We, therefore, focused on approximately 2 kb fragments of the regions where H2AK119ub1 was enriched (*Cdkn1a*: from 29,093,000 to 290,935,000 bp;* Cdkn1c*: from 143,458,997 to 143,460,882 bp; *Cdkn2a*: from 89,290,000 to 89,292,000 bp) and designed four pairs of primers (PP1-PP4) for verification (Figure 5G). Compared with the IgG controls, the quantification by ChIP-qPCR showed enrichment of H2AK119ub1 on the designated *Cdkn1a*/*Cdkn1c* promoter regions (Figure 5H, I). For the *Cdkn2a* promoter, H2AK119ub1 was enriched on three out of four designated regions except the second one (Figure 5J). Therefore, PRC1-targeted H2AK119ub1 was enriched on the promoter of *Cdkn1a*, *Cdkn1c* and *Cdkn2a* to regulate their respective transcription.

### Elevated CDKIs blocked GC proliferation at G1-S transition in dcKO mice

**Figure 6:**
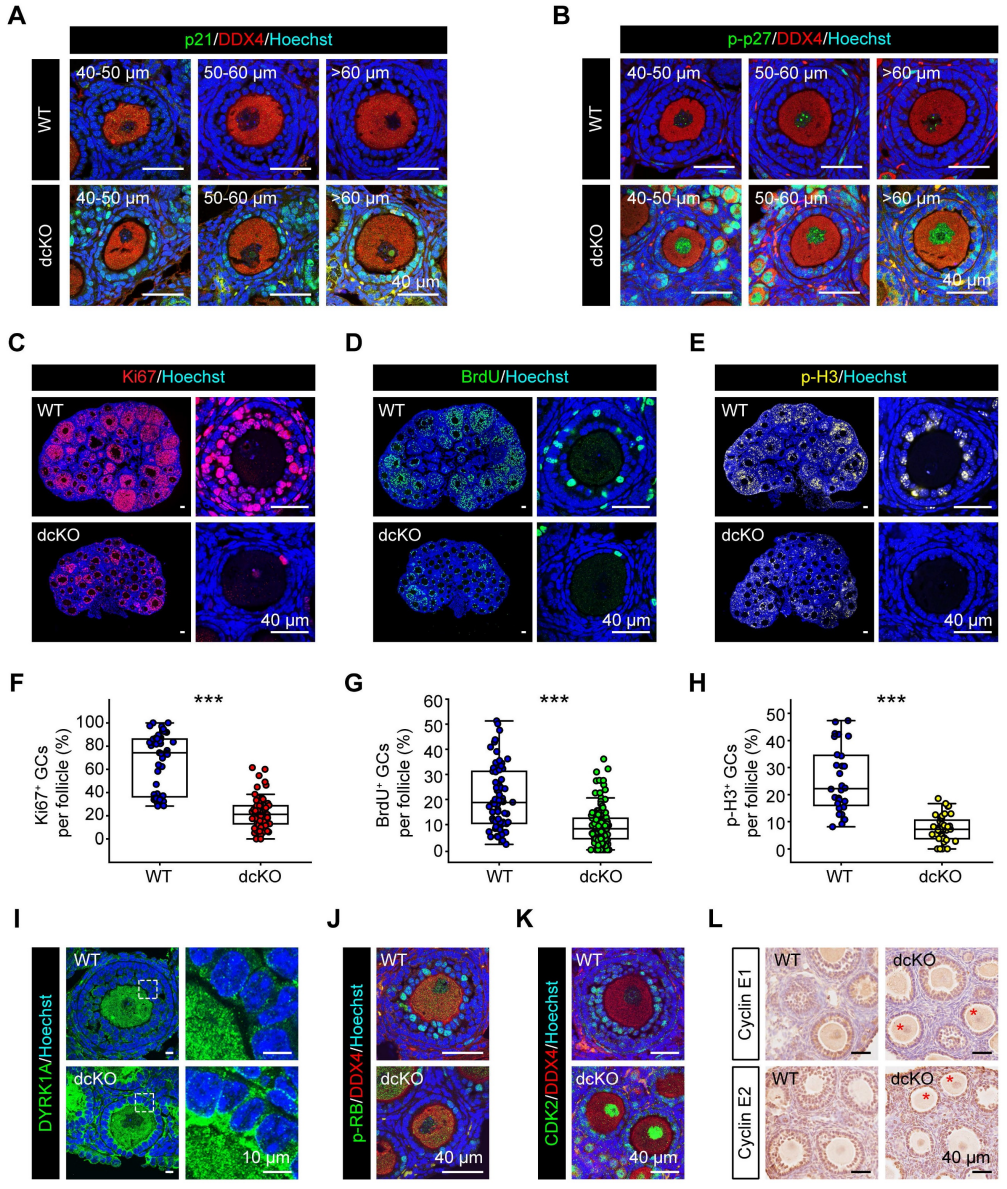
** GC proliferation in dcKO was blocked at G1-S transition due to the elevated CDKIs.** (**A**) p21 staining (green), (**B**) p-p27 staining (green) in 3-week-old ovaries. (**C**, **D**, **E**) Ki67 staining (red), BrdU staining (green), p-H3 staining (yellow, pseudo color) in 3-week-old ovaries respectively. (**F**, **G**, **H**) Percentage of Ki67-, BrdU- and p-H3-positive GCs per follicle. F: n = 48 (WT), n = 82 (dcKO); G: n = 70 (WT), n=138 (dcKO); H: n = 28 (WT), n=40 (dcKO). (**I**) DYRK1A staining (green) of 3-week-old ovaries. Dotted rectangles mark the areas that are enlarged on the right. (**J**) p-RB staining (green), (**K**) CDK2 staining (green) in 3-week-old ovaries. (**L**) Immunohistochemical staining of Cyclin E1 (brown, top), Cyclin E2 (brown, bottom) in 3-week-old ovaries. Asterisks mark abPFs.

We confirmed the above findings by further examining the expression of CDKIs. p21, encoded by *Cdkn1a*, has been reported to be highly expressed in atretic follicles and scattered cells in CLs 25. In dcKO ovaries, p21 exhibited elevated expression in the GCs of abPFs and other developing follicles, while only a few scattered GCs with positive signals were found in AFs of the WT group (Figure 6A, S4D). p27, encoded by *Cdkn1b*, was located in GCs of early-growing follicles with similar mRNA levels between WT and dcKO ovaries (Figure S4D, E). A previous study had identified that a dual-specificity tyrosine phosphorylation-regulated kinase, DYRK1A, phosphorylates p27 on Ser10 to elevate its stability in the nucleus 48. In WT ovaries, the stable form of p27, p-p27, was expressed restrictively in PrFs and PFs (Figure S4B). However, p-p27 was markedly upregulated in the GCs of abPFs (Figure 6B, S4D). Our ChIP-Seq and qPCR data indicated that this might be due to the elevated level of DYRK1A, whose transcription was directly controlled by H2AK119ub1 modification (Figure 6I, S4F, S5B, C).

p21, p27, and p57 were reported to suppress the activity of CDK2/Cyclin E complex to block G1-S phase transition 49, 50. In eukaryotes, the CDK2/Cyclin E complex acts as the restriction point to hyper-phosphorylate and inactivate retinoblastoma protein (RB), the key factor in the transition of G1 to S phase 49-52. In our study, Cyclin E1/E2 levels in abPF GCs were similar to those in SFs of the WT group, whereas the CDK2 expression was reduced (Figure 6K, L, S4H). Also, the p-RB level was much lower in abPFs despite its normal expression in PFs of dcKO ovaries (Figure 6J, S4C, G). Besides, the expression levels of two p-RB-targeted factors, E2F1 and PCNA 53, were greatly decreased (Figure S4K, L). Therefore, the damaged PRC1 resulted in an abnormal expression of CDKIs and perturbed the molecular network of restriction point in G1-S transition.

The termination of GC proliferation in abPFs of dcKO mice was confirmed by examining specific biomarkers, including Ki67-labeled G1-M phase, BrdU-labeled S phase, and p-H3-labeled G2-M phase. As expected, the proportions of GCs with Ki67-, BrdU-, and p-H3-positive signals were decreased in abPFs (Figure 6C-H). The induction of cell apoptosis or senescence by overexpressed CDKIs is well established 54. However, the typical characteristics of either apoptosis (γH2AX, cleaved-Caspase3 and TUNEL) or senescence (SA-β gal) in dcKO ovaries were not detected (Figure S4I, J, M, N). Hence, our results confirmed that the PRC1 damage mainly caused anomalous GC proliferation and not elevated apoptosis or senescence. Thus, PRC1 suppressed CDKIs in GCs during the G1-S phase transition of the cell cycle.

### Activating p21/p27 expression *in vitro* caused a similar phenotype with dcKO mice

**Figure 7:**
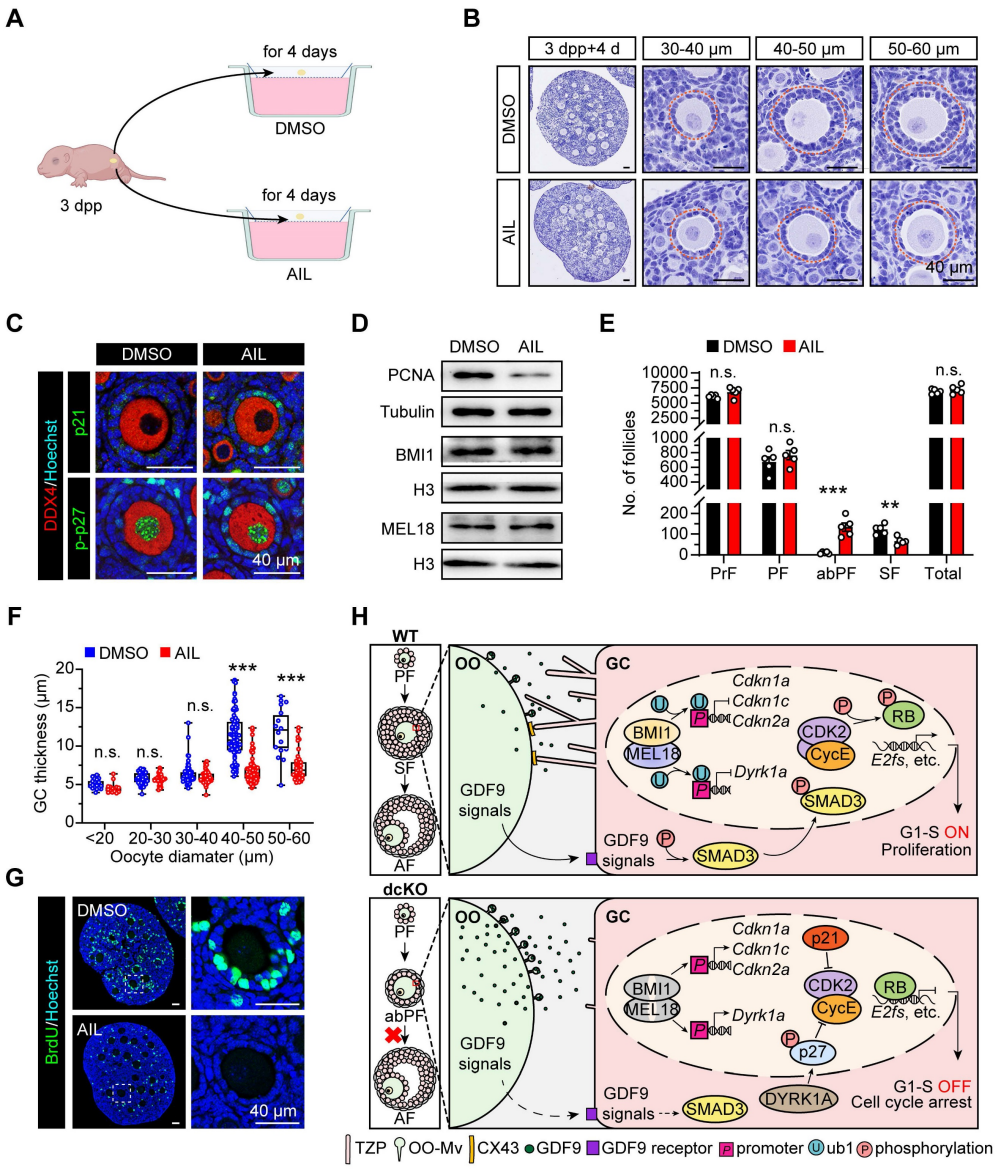
** Upregulating p21/p27 *in vitro* duplicated similar phenotype with abPFs.** (**A**) Schematic diagram showing the strategy of ovary culture *in vitro*. The DMSO group is set as a control. (**B**) Hematoxylin staining showing the follicular morphology of cultured ovaries. Dotted lines mark follicles. (**C**) p21 staining (green, top) and p-p27 staining (green, bottom) in cultured ovaries. (**D**) PCNA, BMI1 and MEL18 protein levels. (**E**) Number of follicles in cultured ovaries. n = 5. (**F**) GC thickness of follicles. n = 204 follicles (DMSO), n=200 follicles (AIL). (**G**) BrdU staining (green) in cultured ovaries. (**H**) Working model of PRC1 in GCs.

Multiple CDKIs were upregulated in dcKO ovaries, making it difficult to rescue the defects *in vivo*. We, therefore, increased the expression levels of CDKIs *in vitro* by specific activators to verify whether they were the key targets of PRC1 to regulate folliculogenesis. Compared with the WT or single-null mice, *p21/p27* double-null mice have larger ovaries and an increased ability of GC proliferation 55. The phenotype is consistent with the GC proliferation defects related to the upregulated p21 and p-p27 in dcKO mice. Therefore, *in vitro* assays were performed applying Ailanthone (AIL) 56, one of the specific activators of p21/p27 expression. To detect the transition from PFs to SFs, 3 dpp ovaries were cultured for 4 days with DMSO or AIL treatment (Figure 7A).

In the AIL group, both* Cdkn1a* and *Cdkn1b* mRNA levels were increased (Figure S6A). As expected, the BMI1/MEL18 expression was similar to the DMSO group (Figure 7D, S6B). Intriguingly, folliculogenesis was blocked by AIL treatment as more abPFs were observed, and p21 and p-p27 were highly expressed in GCs (Figure 7B, C). This defect was consistent with the dcKO mice phenotype. Once the oocyte diameter was larger than 40 μm in size, the GC layer became thinner than that in the DMSO group, and the oocyte was typically enclosed by one-layer cubical GCs (Figure 7B, F). In response to AIL treatment, the number of abPFs was increased while the number of SFs was decreased significantly (Figure 7E, S6C). However, the activation rate of PrFs did not change (Figure S6D), implying that the transition of primary-to-secondary follicles instead of primordial-to-primary activation was the major consequence induced by AIL. Further, the PCNA expression level and BrdU-positive GC proportions in abPFs were decreased (Figure 7D, G, S6B, E). These findings provided evidence that PRC1 controlled GC proliferation by regulating CDKIs.

## Discussion

The transition of primary-to-secondary follicles represents the initiation of folliculogenesis, which involves oocyte growth, GC proliferation, and mutual crosstalk between the oocyte and the surrounding GCs 42, 57. Well-established SFs are essential for gonadotropin-induced responsiveness and oocyte maturation. In our study, the PRC1 function was eliminated by double deletion of *Bmi1*/*Mel18* and resulted in the reduction of H2AK119ub1. During the transition from PFs to SFs, dysfunctional PRC1 blocked GC proliferation by activating CDKI expression due to decreased H2AK119ub1 levels. Concomitantly, the physical interaction and paracrine regulation of crosstalk were impaired, triggering the blockage of early folliculogenesis and eventually leading to female infertility (Figure 7H).

The incidence of POI has risen to 3.7% in females, threatening the health and life of patients 58. A recent large-scale sequencing identified mutations in 1,030 POI genomes, elevating the contribution of genetic variants to 23.5% of POI cases 2. However, most of the pathogenic variants remain underdetermined. In this study, a 17-year-old patient who never had a menstruation period since puberty was selected for assessment. This reproductive defect was more serious than that of her mother and grandmother, suggesting that the genetic mutants in this patient were dominant. Although WGS data identified the *MEL18* mutant, there was little difference in ovarian fertility between the mcKO and WT groups in our mouse models. A recent study reported that the deletion of *Mel18* terminated female fertility at 10 months with fewer pups 34. However, this reproductive damage was still less significant than the clinical characteristics observed in the patient in the present study. The disparity between the mouse models and the patient in our study might be due to the interactions between other genetic defects, highlighting the heterogeneity of clinical POI and the complex pathogenicity of PRC1.

Several findings in our study suggested the complexity of factors inducing accelerated follicular exhaustion and infertility in dcKO females. First, the delayed GC proliferation might trigger hormone abnormalities and endocrine disorders, aggravating the defects in folliculogenesis. Second, accumulated CDKIs in GCs lead to cell senescence and finally trigger follicle loss. Although there were no obvious senescence signals in ovaries at 3 weeks, elevated β-gal staining in the GCs was noticed in 2-month-old dcKO ovaries (Figure S2L, M). In contrast, these signals were only found in the ovarian stromal region in the WT group. Besides, the damaged physical interaction between oocytes and GCs possibly facilitates follicle loss. Furthermore, decreased TZPs were reported as a novel defect of reproductive aging 43, indicating that the TZP reduction in dcKO ovaries impairs folliculogenesis and even accelerates ovarian aging.

Previous studies reported the elimination of the PRC1 function by deleting E3 ubiquitin ligase* Ring1a/b* 30-32. However, our study implicated PRC1 structural subunits in determining its catalytic ability. Although the phenotype of mcKO mice was not critical, double deletion of *Bmi1*/*Mel18* identified the significance of PRC1 in female reproduction, comparable to defects in hormone levels and fertility in POI patients. A previous study showed that *Bmi1*-null mice were infertile due to the oxidative stress in ovaries, and oocytes lacking BMI1 failed to develop into 2-cell stage embryos following *in vitro* fertilization 59. However, bcKO mice in our study maintained normal folliculogenesis at 8 months, indicating that BMI1 was more crucial in oocytes than GCs. p16 and p19 are the most canonical loci targeted by BMI1 or MEL18 60-62. Interestingly, our study showed that p21 and p-p27 also played vital roles in the PRC1-mediated proliferation of GCs, confirmed by a series of *in vitro* assays. Also, different expression patterns of p21/p-p27 in early folliculogenesis indicated that PRC1 regulated CDKI levels in multiple ways. For regulatory manners, PRC1 controlled CDKI expression by regulating its transcription (like *Cdkn1a*, *Cdkn1c,* and *Cdkn2a*) or regulating the activator of CDKIs (like *Dyrk1a*), which were dependent on H2AK119ub1 enrichment on the promoters. For physical function, PRC1 was either to clean up the CDKIs accumulated from PrF stage (like p-p27), or to prevent the CDKIs expressed in degraded or senescent cells to elevate prematurely (like p21) 25, 63.

Mouse models have previously been described in which the follicular blockades, including WNT signaling damage and* Gdf9*- or *Cx43*-null mice, resulted in a phenotype similar to the dcKO mice 24, 46, 64. In WNT signaling damage models, like *R-spondin2*-mutant and *Catenin beta-1* (*Ctnnb1*) *^f/f^*; *Wt1-CreER^T2^* mice, folliculogenesis was regulated by CTNNB1, the classic mediator of WNT signals 64. However, CTNNB1 expression was intact in abPFs, implying a lack of interaction between PRC1 and WNT signals (Figure S3F). GDF9 is one of the most vital OSFs to accelerate GC proliferation in preantral follicles 65, 66, required to generate TZPs and rescue GC proliferation defects in *Cx43-null* mice 43, 44, 47. Mice lacking *Gdf9* are infertile due to the blockage at type 3b follicles, which have high similarities with abPFs, including proliferation and apoptosis failure 25, 67. Type 3b follicles maintained CX43 expression, which was lacking in abPFs 47. As per the GC RNA-seq data, PRC1 did not regulate the transcription of *Cx43*, TZP-related genes, like *Daam1*, *Fscn1* and *Myo10*, and GDF9-related genes like *Smad3*, *Alk5,* and *Bmpr2* 43, 68, 69 (Data not shown). However, the intercellular crosstalk was apparently disturbed in dcKO mice. It may be attributed to the reduced responsiveness to GDF9 induced by the developmental defects in mutant GCs.

Our data showed that despite the normal expression of GDF9 signal receptors ALK5 and BMPR2, SMAD3 was not phosphorylated (Figure S3G). This defect possibly blocked TZP generation, accelerated GDF9 secretion, and OO-Mv formation in a negative feedback loop 43. Thus, the potential relationship between PRC1 and p-SMAD3 needs to be further investigated. Additionally, the absence of CX43 in abPFs probably aggravates the lack of response to GDF9, even though the mechanism remains unclear 47. However, despite the increased density of OO-Mv in abPFs, the possibility of potential defects in the delivery of GDF9 cannot be excluded 44. Our findings provided evidence of a complex interactional network between paracrine signaling, intercellular communication, and GC proliferation. Additionally, PRC1 in GCs was essential for responding to oocyte-derived GDF9 signals.

In conclusion, we demonstrated that PRC1 is required for GC proliferation in early folliculogenesis. Double deficiency of BMI1/MEL18-induced dysfunctional PRC1 blocks the GC mitotic cell cycle by elevated CDKIs, impairing the primary-to-secondary follicular transition and female fertility. Our findings provide insights into the epigenetic mechanisms of early follicular development and contribute to understanding the pathology of PRC mutation-related POI. Future studies characterizing PRC1 and its subunits may contribute to elucidating the potential mechanisms of POI in translational research and clinical therapy.

## Supplementary Material

Supplementary figures and table.
