# Supplementary material for: Polycomb repressive complex 1 modulates granulosa cell proliferation in early folliculogenesis to support female reproduction
Source: Theranostics. 2024 Jan 27;14(4):1371–89. doi: 10.7150/thno.89878 (PMC10879878; doi:10.7150/thno.89878)
Supplement: Supplementary file 1 — Supplementary figures and table. [file thnov14p1371s1.pdf]

Genotyping primers

Gene	Forward sequence	Reverse sequence
Bmi1	GCGATTGATAACAGGACAGTCA	GGGTAGGGAGGAAAGAAGCA
Mel18	TGTGGTTTGTTGGTGGACAGC	CTCGGCAGGTAAAGGAAGACTC
Foxl2-Cre	TGCTTCTGTCCGTTTGC	CCACCGTCAGTACGTGAG

RT-qPCR primers

Gene	Forward sequence	Reverse sequence
Actb	CATTGCTGACAGGATGCAGAAGG	TGCTGGAAGGTGGACAGTGAGG
Gdf9	TCACCTCTACAATACCGTCCGG	GAGCAAGTGTTCCATGGCAGTC
Cdkn1a	TGTCGCTGTCTTGCACTCG	GACCAATCTGCGCTTGGAGT
Cdkn1b	AGCAGTGTCCAGGGATGAGGAA	TTCTTGGGCGTCTGCTCCACAG
Cdkn1c	AGCTGAAGGACCAGCCTCTCTC	ACGTCGTTCGACGCCTTGTTCT
Cdkn2a	GAACTCTTTCGGTCGTACCC	CGAATCTGCACCGTAGTTGA

ChIP-qPCR primers

Gene		Forward sequence	Reverse sequence
Cdkn1a	PP1	ACAGACGACCTTTCGGTTTGTGCCT	ACAGGGATGAGGAGAATGGAAATAG
	PP2	GCTGTCCTGGAACTCACTTTGTAGA	GACCTCAGACTCACTATGTAGCCAA
	PP3	GGAAGGAGAAGGAGCAGTCCATGTT	TAATGCTAGAGTGGCGTTGGACAGG
	PP4	AGAATGAATGCCAGACTCTCCAAGC	TGGAAAAATTTGTTTAAAGTTAGCG
Cdkn1c	PP1	CACAAAAAGGAGACAGAGGGTTAAG	AACAGTAGAGAGAGGTGGTCTTTGC
	PP2	AGGGTTTATTAGCTTACACTTTCCA	TAATTAGTGAACAAATGGGGAGGGC
	PP3	TCCCTGAGGAGTTAAAGCAGTCTAC	ACAGAATACACCAAGTGGGAGGCAT
	PP4	AAGCTGGAGGGTGAAGGGTGTATGT	AGGTCCACCATCTGTCCCTCTCTGT
Cdkn2a	PP1	ACATGCATACCATAGACTGGGAGAC	TTGAGAATCAGGGCACTTCCTTAAT
	PP2	AACCTCCCTAAACCTCTTCATCTAA	TTTAGAGCAGTGGTTCTCAGCCTTC
	PP3	AAACATTTAGCTCTGCCTGTACGTG	AGTGAAACAGTATTTGCTGGGCGTG
	PP4	GCAGGAAGCTATAGGTTGTTCTCAC	TTCTTGAGTCATAGACCACAAATAA
Dyrk1a	PP1	AGTAAAACCCTAACTAAGACAGATT	AAGTGCTAGGATTATAAACACATGC
	PP2	TGGGAGGTGGAGGTAGGAGGATCAG	CTCTGTATGTCTGCTGGAGAAAGGA
	PP3	ACCCAGAAGACAACACTGTTCACAA	ACTCCCGTAGTGGGCTCTTCTCCTC

Table. S1 Primer sequences of genotyping, RT-qPCR and ChIP-qPCR.

A

Clinical characteristics

	Ref values	patient (III)
Age (years)	—	17
Gender	—	female
Karyotype	—	46, XX
Diagnosis of disease	—	POI

Hormone analysis

FSH (IU/mL)	25.80 - 134.80 (menopause)	73.66
LH (IU/mL)	7.70 - 58.50 (menopause)	54.90
Estrogen (pmol/mL)	< 18.40 - 505.00 (menopause)	< 18.35

B

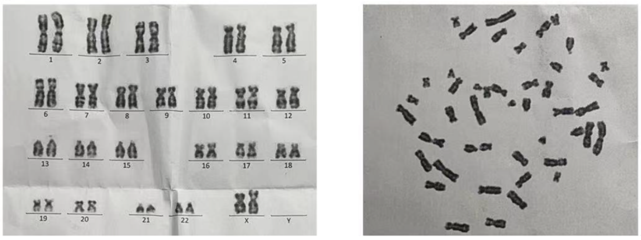

Figure S1. The clinical data of the POI patient.

(**A**) Clinical characteristics of the POI patient. (**B**) Karyotype analysis.

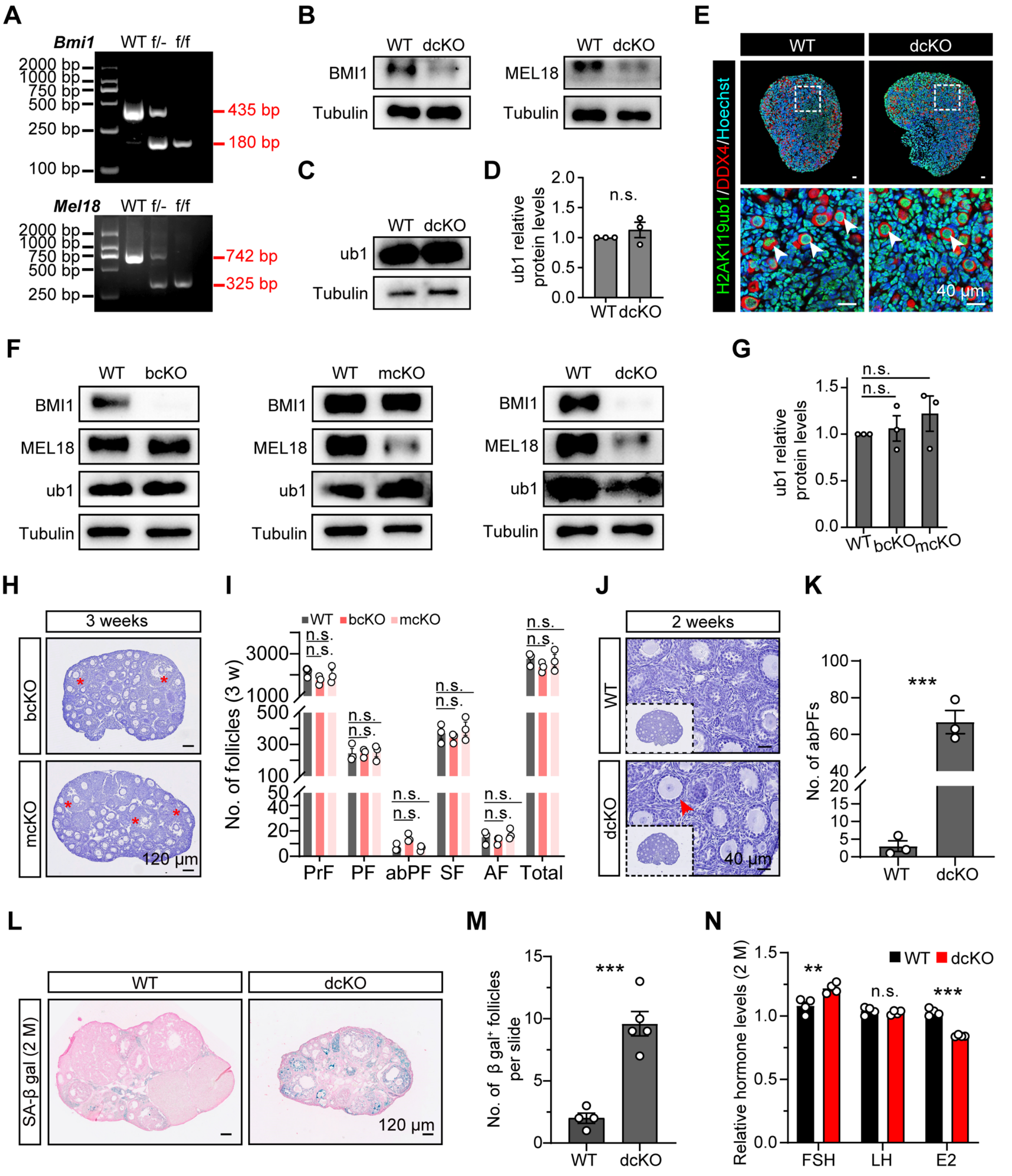

Figure S2. Conditional knockout efficiencies and the phenotypes at given ages.

(**A**) Genotypic identification. (**B**, **C**) Efficiency of Cre-LoxP system in 19.0 dpc ovaries. (**D**) Relative protein levels of H2AK119ub1 in 19.0 dpc ovaries by grey scanning. n = 3. (**E**) H2AK119ub1 staining (green) in 19.0 dpc ovaries. Dotted rectangles mark the follicles that are enlarged on the bottom. Arrowheads mark PrFs. (**F**) Efficiency of Cre-LoxP system in 3-week-old ovaries. (**G**) Relative protein levels of H2AK119ub1 by grey scanning. n = 3. (**H**) Hematoxylin staining of 3-week-old bcKO and mcKO ovaries. Asterisks mark AFs. (**I**) Number of follicles in 3-week-old bcKO/mcKO ovaries. n = 3. (**J**) Hematoxylin staining of 2-week-old ovaries. Arrow marks abPF. (**K**) Number of abPFs in 2-week-old ovaries. n = 3. (**L**) SA-β gal staining showing senescent cells (blue) in 2-month-old ovaries. Pink stanning showing nucleus with nuclear fast red. A 14-month-old WT ovary is set as a positive control. (**M**) Number of follicles with β gal-positive GCs per slide in 2-month-old ovaries. n = 4 (WT), n = 5 (dcKO). (**N**) Relative hormone levels of FSH, LH and E2 in 2-month-old mice serum. n = 4.

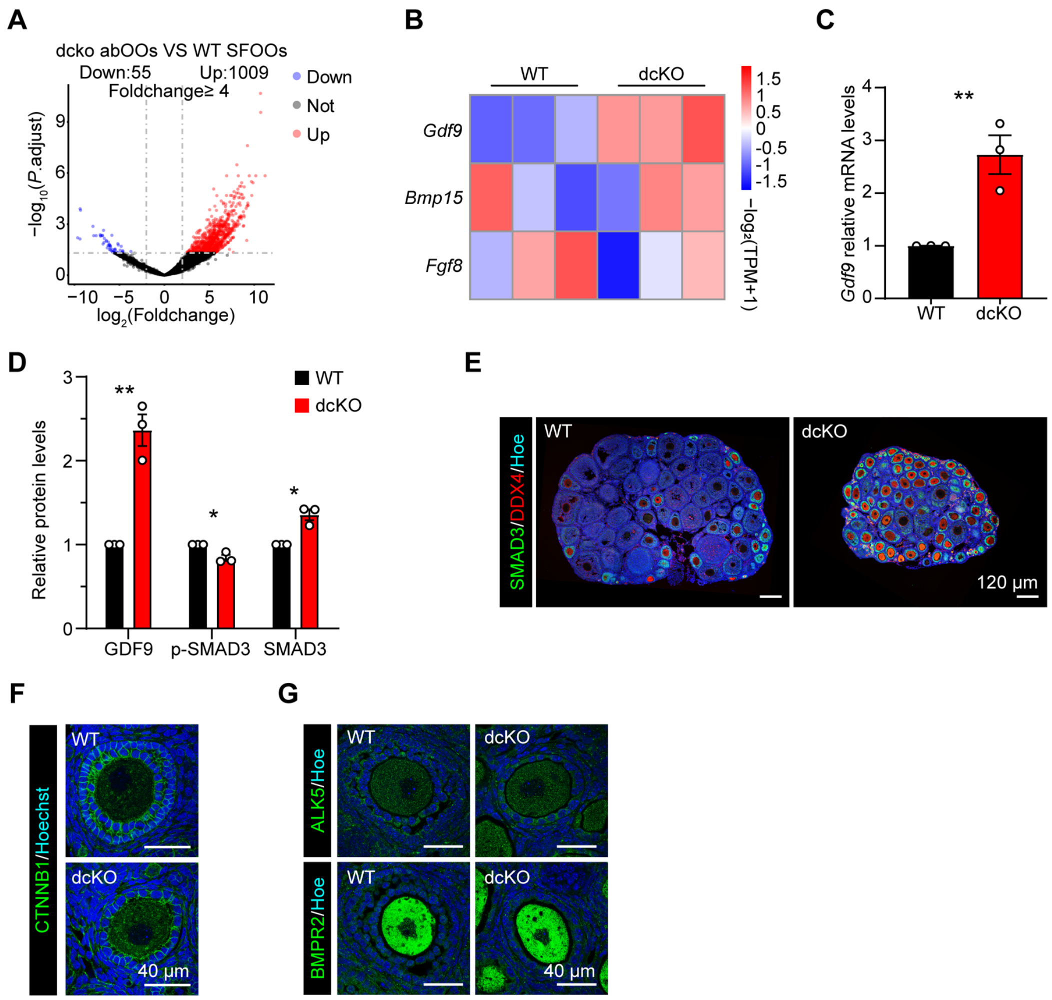

Figure S3. GDF9-SMAD3 signaling pathway was blocked in dcKO mice.

(**A**) Volcano plots showing the comparison of transcriptomes between the oocytes of early SFs in WT and those of abPFs in dcKO at 3 weeks. (**B**) Heatmaps of OSFs according to oocyte RNA-seq data. (**C**) *Gdf9* relative mRNA levels in 3-week-old ovaries. n = 3. (**D**) Relative protein levels of GDF9, p-SMAD3 and SMAD3 in ovaries by grey scanning. n = 3. (**E**) SMAD3 staining (green) in 3-week-old ovaries. (**F**) CTNNB1 staining (green), (**G**) ALK5 staining (green, top), BMPR2 staining (green, bottom) in 3-week-old ovaries.

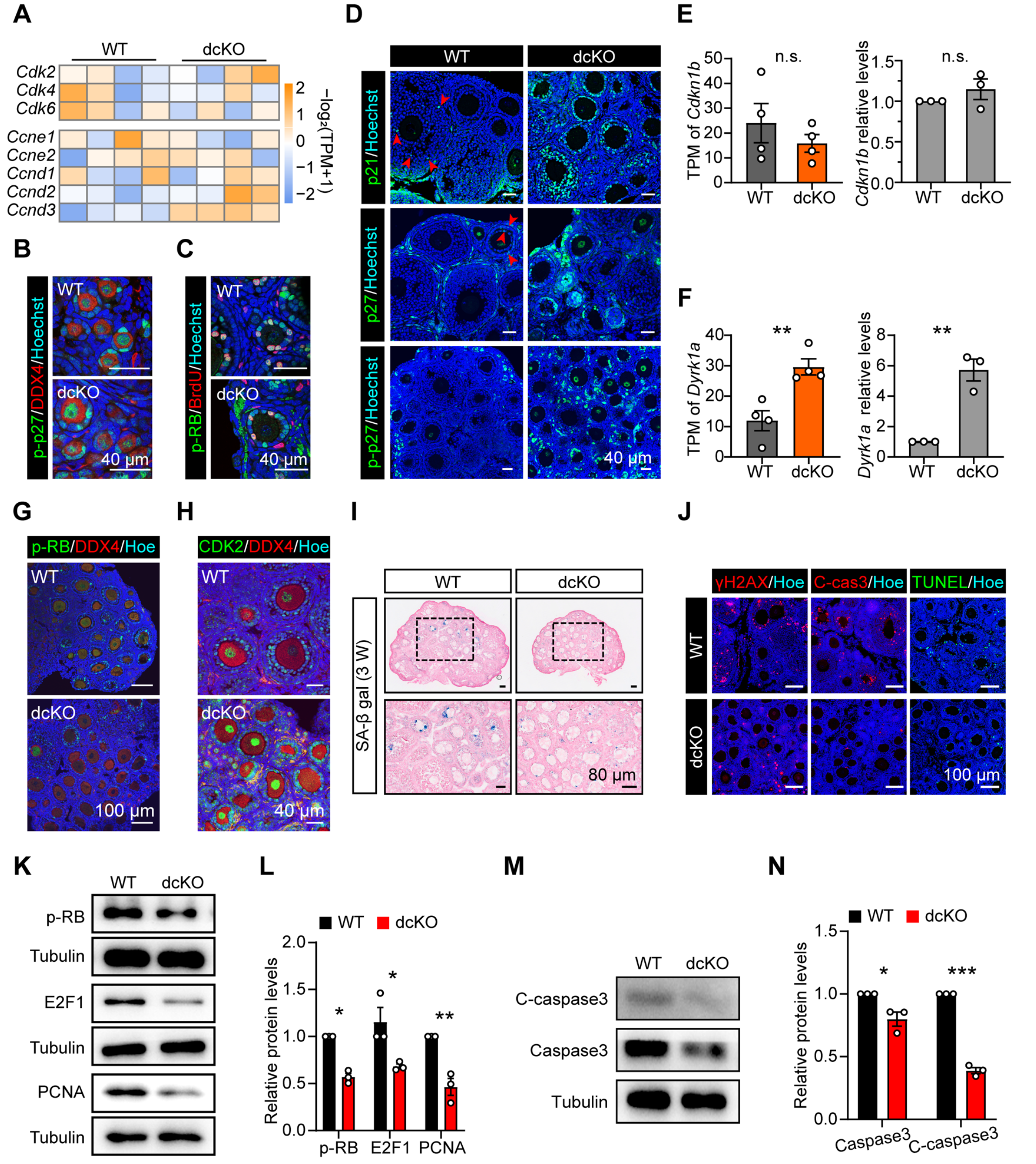

Figure S4. GC proliferation, instead of apoptosis or senescence, was impaired in dcKO mice.

(**A**) Heatmap of CDKs and Cyclins according to GC RNA-seq. (**B**) p-p27 staining (green), (**C**) p-RB staining (green) in PFs. (**D**) p21 staining (green, top), p27 staining (green, medium), p-p27 staining (green, bottom) in 3-week-old ovaries. Arrowheads mark the GCs with positive staining. (**E**, **F**) TPM in GCs and relative mRNA levels of *Cdkn1b* and *Dyrk1a* in 3-week-old ovaries. n = 3 (RT-qPCR). (**G**) p-RB staining (green), (**H**) CDK2 (green) in 3-week-old ovaries. (**I**) SA-β gal staining showing senescent cells (blue) in 3-week-old ovaries. Dotted rectangles mark the follicles that are enlarged at the bottom. (**J**) γh2AX staining (red, left), C-cas3 staining (red, medium), TUNEL staining (green, right) in of 3-week-old ovaries. C-cas3, cleaved-caspase 3. (**K**, **L**) p-RB, E2F1 and PCNA protein levels and relative protein levels by grey scanning in 3-week-old ovaries. n = 3. (**M**, **N**) C-caspase 3, Caspase 3 protein levels and relative protein levels by grey scanning in 3-week-old ovaries. n = 3.

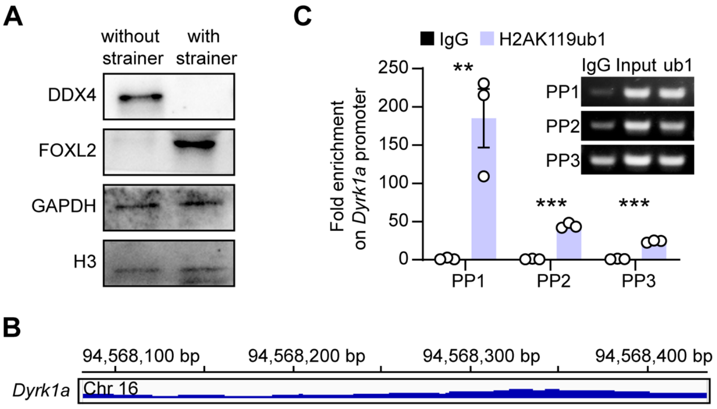

Figure S5. Data related to ChIP.

(**A**) Efficiency of separating GCs by cell strainers. (**B**) IGV data showing H2AK119ub1 is enriched on the promoter of *Dyrk1a*. (**C**) Enrichment degrees of H2AK119ub1 on the *Dyrk1a* promoter.

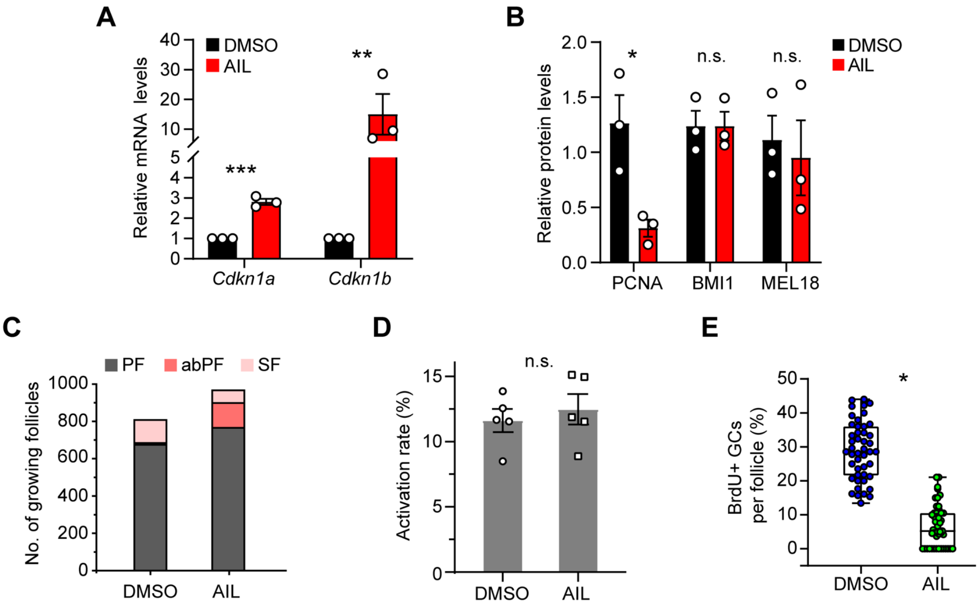

Figure S6. Data related to cultured ovaries *in vitro*.

(**A**) Relative mRNA levels of *Cdkn1a* and *Cdkn1b*. n = 3. (**B**) Relative protein levels of PCNA, BMI1 and MEL18 by grey scanning in cultured ovaries. n = 3. (**C**) Count of PFs, abPFs and SFs in cultured ovaries. Each colored column indicates the mean value. (**D**) PrF activation rate in cultured ovaries. n = 5. (**E**) Percentage of BrdU-positive GCs in follicles of cultured ovaries. n = 46 (DMSO), n = 47 (AIL).
